# Evolutionary selection across the nuclear hormone receptor superfamily with a focus on the NR1I subfamily (vitamin D, pregnane X, and constitutive androstane receptors)

**DOI:** 10.1186/1478-1336-3-2

**Published:** 2005-09-30

**Authors:** Matthew D Krasowski, Kazuto Yasuda, Lee R Hagey, Erin G Schuetz

**Affiliations:** 1 Department of Pathology, Children's Hospital of Pittsburgh, 5834 Main Tower, 200 Lothrop Street, University of Pittsburgh, Pittsburgh, PA, 15213 USA; 2 Department of Pharmaceutical Sciences, St. Jude Children's Research Hospital, Memphis, TN, 38105 USA; 3 Department of Medicine, University of California, San Diego, CA, 92093, USA

## Abstract

**Background:**

The nuclear hormone receptor (NR) superfamily complement in humans is composed of 48 genes with diverse roles in metabolic homeostasis, development, and detoxification. In general, NRs are strongly conserved between vertebrate species, and few examples of molecular adaptation (positive selection) within this superfamily have been demonstrated. Previous studies utilizing two-species comparisons reveal strong purifying (negative) selection of most NR genes, with two possible exceptions being the ligand-binding domains (LBDs) of the pregnane X receptor (PXR, NR1I2) and the constitutive androstane receptor (CAR, NR1I3), two proteins involved in the regulation of toxic compound metabolism and elimination. The aim of this study was to apply detailed phylogenetic analysis using maximum likelihood methods to the *entire *complement of genes in the vertebrate NR superfamily. Analyses were carried out both across all vertebrates and limited to mammals and also separately for the two major domains of NRs, the DNA-binding domain (DBD) and LBD, in addition to the full-length sequences. Additional functional data is also reported for activation of PXR and the vitamin D receptor (VDR; NR1I1) to gain further insight into the evolution of the NR1I subfamily.

**Results:**

The NR genes appear to be subject to strong purifying selection, particularly in the DBDs. Estimates of the ratio of the non-synonymous to synonymous nucleotide substitution rates (the ω ratio) revealed that only the PXR LBD had a sub-population of codons with an estimated ω ratio greater than 1. CAR was also unusual in showing high relative ω ratios in both the DBD and LBD, a finding that may relate to the recent appearance of the CAR gene (presumably by duplication of a pre-mammalian PXR gene) just prior to the evolution of mammals. Functional analyses of the NR1I subfamily show that human and zebrafish PXRs show similar activation by steroid hormones and early bile salts, properties not shared by sea lamprey, mouse, or human VDRs, or by *Xenopus laevis *PXRs.

**Conclusion:**

NR genes generally show strong sequence conservation and little evidence for positive selection. The main exceptions are PXR and CAR, genes that may have adapted to cross-species differences in toxic compound exposure.

## Background

Nuclear hormone receptors (NRs) are ligand-activated transcription factors that work in concert with co-activators and co-repressors to regulate gene expression [1-3]. NRs share a modular domain structure, which includes, from N-terminus to C-terminus, a modulatory A/B domain, the DNA-binding domain (DBD; C domain), the hinge D domain, the ligand-binding domain (LBD; E domain) and a variable C-terminal F domain that is absent in some NRs [3]. Examples of ligands for NRs include a range of endogenous compounds such as steroid hormones, thyroid hormone, and retinoids [3,4]. A few NRs, such as the 'xenobiotic sensors' pregnane X receptor (PXR, NR1I2) and constitutive androstane receptor (CAR or NR1I3), are activated by structurally diverse exogenous ligands [5-7].

The NR superfamily in mammals is composed of approximately 50 functional genes, with 48 genes in humans, 47 in rats, and 49 in mice [8]. Bony fish have a somewhat larger complement of NR genes due to gene duplication, exemplified by the 68 NR genes found in the genome of the pufferfish *Fugu rubripes *[9]. The current official nomenclature for NRs divides the superfamily into 7 families (NR0-6) [10,11]. The NR0 family, represented in humans by DAX-1 (dosage-sensitive sex and AHC critical region on the X chromosome; NR0B1) and SHP (small heterodimer partner; NR0B2) are unusual in essentially being 'domain singletons' that lack a DBD [12,13]. NRs have been the focus of a number of evolutionary studies including detailed investigations into the origins of the superfamily [11,14-16] and the development of ligand selectivity by the sex and adrenocortical steroid hormone receptors [17-20].

A major focus of molecular phylogenetics has been a search for evidence of positive selection (molecular adaptation) [21]. A variety of computational techniques have been developed over the last several decades to detect nucleotide variation between different genes suggestive of positive selection [21,22]. For comparisons within coding regions, the most common approach is to compare nucleotide variation that is non-synonymous (i.e., changes amino acid sequence encoded for by codons) or synonymous (does not changes amino acid sequence). Synonymous variation is considered to be neutral, an assumption which is generally true although there are exceptions [23]. The ratio of the rate of non-synonymous versus the rate of synonymous nucleotide variation (i.e., how many non-synonymous or synonymous changes have occurred in comparison to the total number of non-synonymous or synonymous changes possible; d_N_/d_S _or ω) provides some indication into selective forces acting on a given gene. For most gene comparisons, ω is less than one, often less than 0.1, reflective of negative or purifying selection to maintain a conserved amino acid sequence. ω = 1 reflects neutral selection (a ratio that would be expected for a non-functional pseudogene) while ω > 1 suggests positive selection. A large-scale comparison of 3,595 groups of homologous genes revealed that less than 0.5% had ω ratios greater than 1, with many of these genes being found in microorganisms [24]. Given that comparisons between full-length gene sequences rarely result in ω ratios greater than 1, techniques have been developed to detect sub-populations of codons that have elevated ω ratios. Different mathematical approaches have been applied to achieve this goal, including maximum likelihood [25,26] and Bayesian [27] methods. In this study, we employed the PAML (Phylogenetic Analysis by Maximum Likelihood) software, developed by Yang and colleagues [28], as this methodology is robust and has an extensive published literature associated with its application in biomedical research [21].

Most of the NR genes are strongly conserved between vertebrate species. Not surprisingly, previous studies utilizing two-species comparisons between human, mouse, and rat or humans, chimpanzee, and mouse genomes revealed that the NR genes are in general subject to negative selection [8,29], with only a few possible exceptions such as the LBDs of PXR and CAR [8,30]. A more detailed phylogenetic analysis of PXR, CAR, and the other member of the NR1I subfamily, the 1,25-(OH)_2_-vitamin D_3 _receptor (VDR; NR1I1), within mammals and across vertebrates, showed ω ratios for the CAR and PXR LBDs markedly higher than that for the VDR LBD [30]. For the PXR LBD analyses within mammals, the ω ratio exceeded one for a sub-population of codons comprising approximately 5% of the total codons in the LBD [30].

Given that a major function of PXR and CAR is to detect toxic endogenous and xenobiotic compounds that likely differ between species [5,6], these two genes may represent unusual examples of NR genes that have undergone positive selection in their LBDs for functional advantage. The aim of this study was to apply detailed phylogenetic analysis to the *entire *superfamily of NR genes in vertebrates to detect possible signatures of positive selection. This phylogenetic analysis, combined with functional analyses of PXRs and VDRs, provides a detailed context into how unusual the nucleotide variation of PXR and CAR is to the rest of the superfamily.

## Results

### Sequences available for phylogenetic analysis

Data from genome sequencing projects (e.g., human, chimpanzee, mouse, rat, dog, chicken, *Xenopus tropicalis*, *Fugu rubripes*, and *Tetraodon nigroviridis*) has greatly increased the number of NR coding sequences publicly available for phylogenetic analysis across vertebrates. The complete set of species and accession numbers for the NR genes analyzed in this study is provided in Additional file 1: Genes used for phylogenetic analysis. Complete nucleotide sequences in PAML format are provided in Additional file 2: Sequences used for phylogenetic analysis by PAML. To improve the power of accurately detecting positive selection and to minimize the risk of false positives, PAML analyses were only performed if sequence data from at least six species from at least six separate genera were available [22,31-33]. Given that some of the sequence data was partial and only contained complete data for the DBD or LBD (and not full-length sequence in those instances), the number of species available for the various analyses for each gene (i.e., full-length, DBD only, and LBD only) differs in some cases. The number of sequences varies widely across the NR superfamily, mainly because some receptors have been more intensively studied than others. For example, 33 full-length sequences are available for analysis of the estrogen receptor-α (ERα, NR3A1), while only 6 are available for estrogen-related receptor-β (ERRβ, NR3B2).

### PAML analyses of the NR superfamily

The PAML analyses used correspond to models M0, M3 (with ncatG = 2, 3, 4, where ncatG is the number of populations of codons with distinct ω ratios), M7, and M8 within the PAML software (see Materials and Methods) [25,26]. Models M0 and M3 are 'discrete' in that they assign codons to population(s) of distinct ω ratios. For instance, an analysis of a particular gene may assign 95% of codons to an ω ratio of 0.05 (consistent with purifying selection) and the remaining 5% to an ω ratio exceeding 1 (suggestive of positive selection). For each M0/M3 analysis, the 'best minimum model' was determined. An M3 analysis was chosen only if it was statistically superior to the closest simpler model (e.g., M3 with 2 ω ratios versus M0). The ω ratios in the M0 or M3 analyses may be any value 0 or greater. M7 assigns codons within a gene to ω ratios along a β distribution function between 0 and 1 with parameters α and β. Depending on the parameters α and β, the β distribution may have most ω ratios clustered near 0 or be distributed more evenly between 0 and 1. M8 is a model where some codons have ω ratios that fall along a β distribution but the remaining codons form a separate population with a discrete ω ratio that may be any value 0 or greater (including greater than 1). M8 can detect the presence of positive selection (i.e., the extra ω class can be greater than 1) whereas M7 cannot.

### M0 and M3 analyses

**Figure 1:**
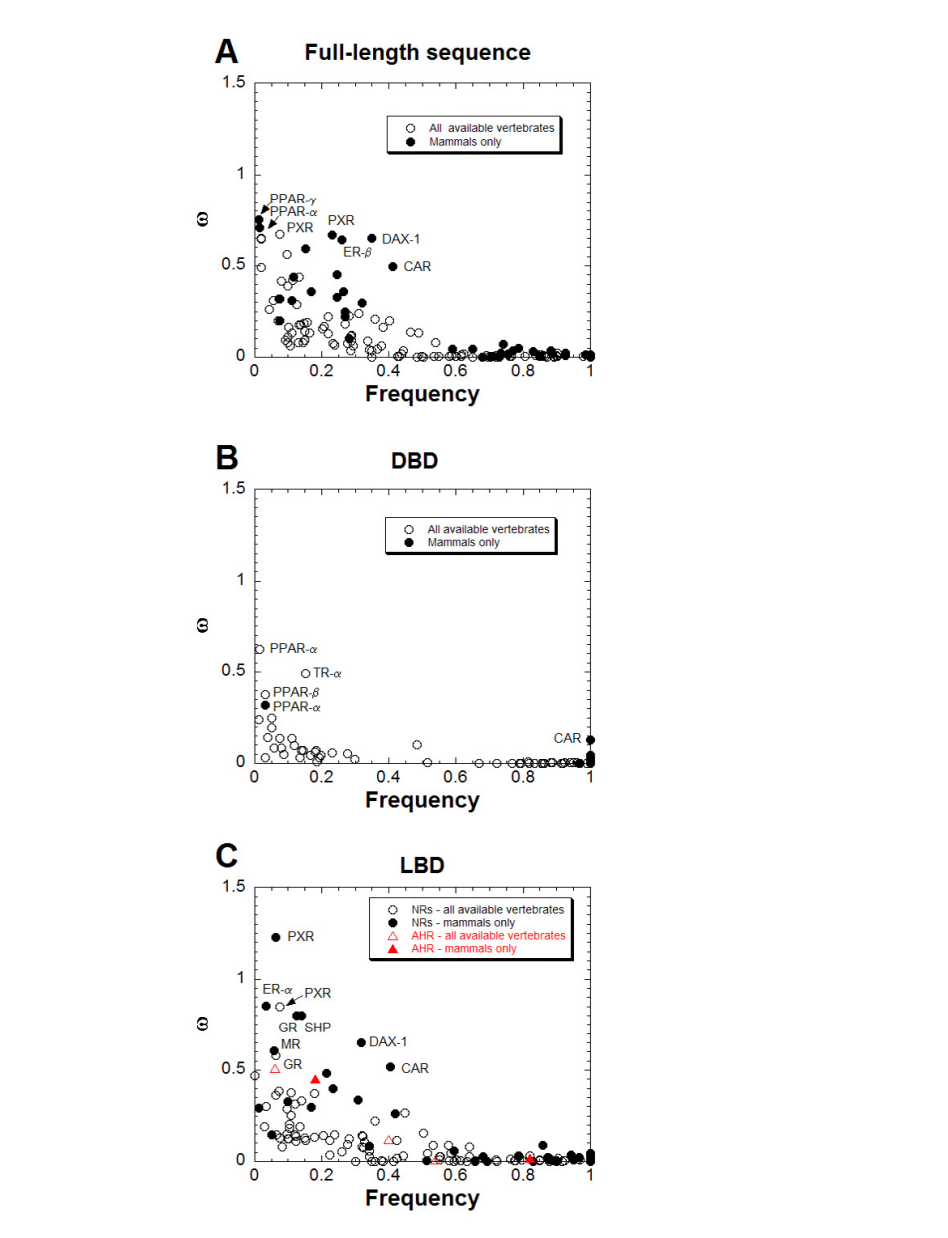
**Summary of PAML discrete ω ratio variation models**. Each point on the plots in **(A)**, **(B)**, and **(C) **corresponds to the frequency and ω ratios for the best minimum model (e.g., M0, M3/ncatG = 2, M3/ncatG = 3, etc.) that provides a statistically superior fit to the data (i.e., a more complex model with additional codon ω ratio classes that does not provide a statistically better fit to the next simplest model is rejected). For example, the analysis of the full-length sequence of NR1A1 (TRα) for all available vertebrate species shows that M3/ncatG = 3 is superior to M3/ncatG = 2 but statistically equivalent to M3/ncatG = 4. Consequently, plotted on Figure 1 are three points for the NR1A1 M3/ncatG = 3 analysis corresponding to frequency and ω ratios for three classes of codons – 80.6% (frequency = 0.806) of codons have an estimated ω ratio of 0.004, 14.9% have an ω ratio of 0.094, and 4.5% have an ω ratio of 0.259. An analysis that shows M0 is the best minimum model will have 100% of codons (frequency = 1.0) with a particular ω ratio. **(A)**, **(B)**, and **(C) **apply to analyses of full-length sequences, DBD only, and LBD only, respectively. The open circles are for analyses of all available vertebrate sequences while the closed circles are for analyses of mammals only. For part **(C)**, the red open and closed triangles represent data for the LBD of the AHR gene (a non-NR gene that encodes a protein with similar function to PXR and CAR).

The complete results for all PAML analyses are provided in Additional file 3: Results of PAML analysis and treefiles. The distribution of frequency and ω ratios for all NR genes is plotted in Figure 1 (parts A, B, and C correspond to full-length sequence, DBD only, and LBD only analyses, respectively; the open circles are for analyses of all vertebrates while the closed circles are for analyses confined to mammals only). Each point on the plots in Figure 1 corresponds to the frequency and ω ratios for the best minimum model that provides a statistically superior fit to the data over the closest simpler model (see legend to Figure 1 for more details).

Overall, the M0/M3 analyses confirm original observations from two-species comparisons that the NR genes are in general subject to strong negative selection, particularly in the DBD [8,29]. The DBDs show lower ω ratios than the LBD or full-length sequences (Figure 1B). Only NR1C1 (peroxisome proliferator-activated receptor-α; PPARα) has a sub-population of codons within the DBD with an estimated ω ratio of greater than 0.5, and this sub-population corresponds to only 1 codon out of 66 analyzed. A number of genes, including NR1B1 (retinoic acid receptor-α; RARα), NR2B1 (retinoid X receptor-α; RXRα), NR3B1 (ERRα), and NR3B2 (ERRβ), show virtually no non-synonymous nucleotide differences between different species in the DBD and have ω ratios close to 0 (< 0.01). NR1A1 (TRα) and NR1I3 (CAR) are somewhat unusual relative to the other NR genes in having at least 15% of codons with an estimated ω ratio of greater than 0.1.

The LBDs of the NR genes clearly show higher ω ratios than the DBDs (Figure 1C). Yet, despite this, the majority of receptors (41 of 48) have ω ratios for all sub-populations of codons less than 0.5. Only PXR genes, analyzed for mammals only, have a sub-populations of codons with an ω ratio greater than 1; this ω class corresponds to 5% of all analyzed codons in the PXR LBD. The analyses for the full-length receptor sequences generally follow the trends seen for the LBD only analysis with minor differences (compare Figure 1A and 1C). To provide another comparison to PXR and CAR, PAML analysis was applied to the LBD of the aryl hydrocarbon receptor-1 (AHR), a non-NR that has a similar function to CAR and PXR, namely to respond to ligands (including xenobiotics) and regulate expression of genes involved in metabolism and elimination of potentially toxic compounds [34]. In contrast to PXR and CAR, the ω ratios associated with the AHR LBD were more similar to the majority of NR genes than to PXR or CAR (see red open and closed triangles in Figure 1C).

### M7 and M8 analyses

For most analyses, M8 was not statistically superior to the neutral model M7. Only 10 of 132 analyses of all vertebrate species and 3 of 65 analyses for mammals-only revealed M8 results statistically superior to M7. None of the M8 analyses identified a sub-population of codons with an ω ratio exceeding 1. Once again, however, analysis did identify a sub-population of codons within the PXR LBD with a high ω ratio relative to other NRs (e.g., for analysis of the full-length PXR receptors for all vertebrates, the M8 analysis found 3.4% of the PXR codons, all within the LBD, with an ω ratio of 0.97).

**Figure 2:**
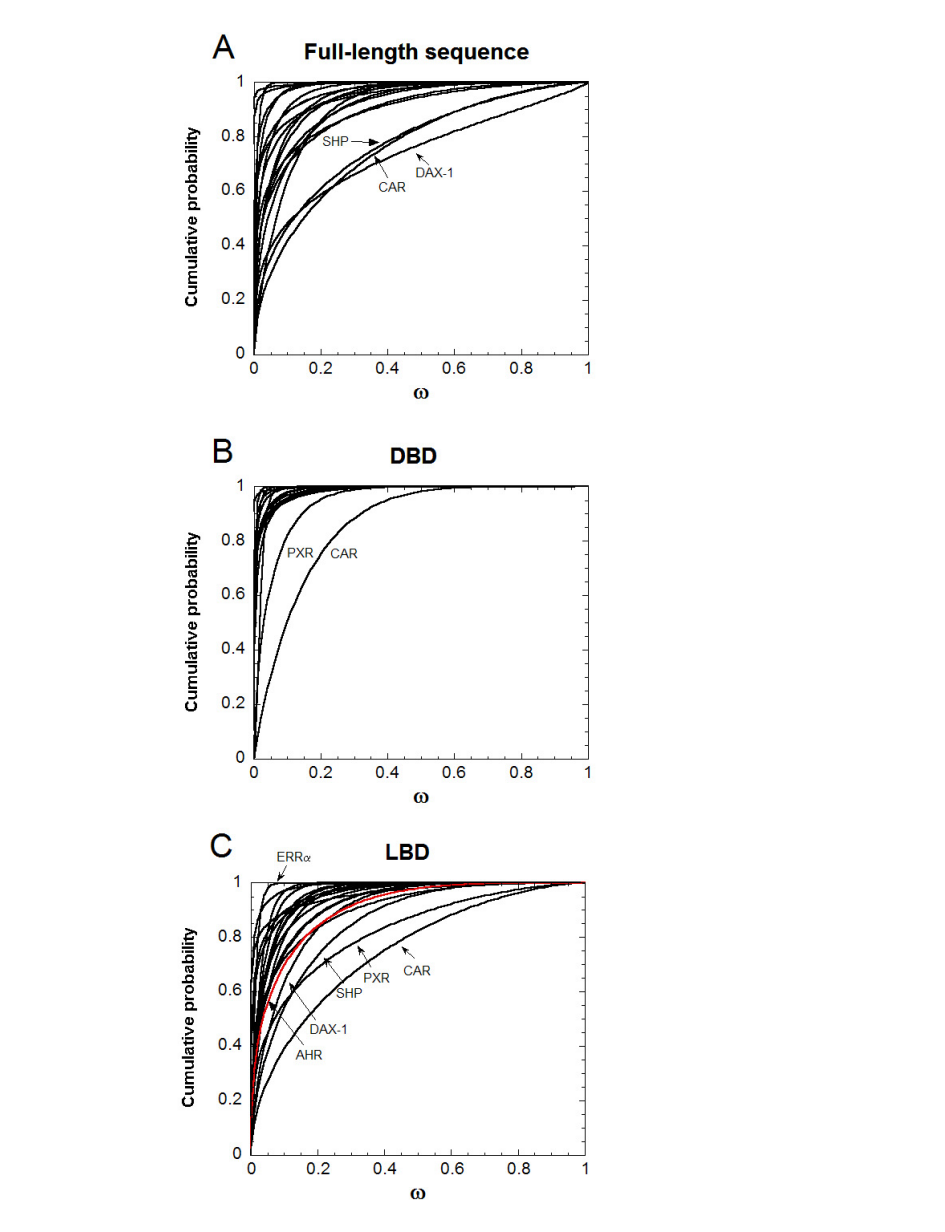
**Summary of PAML β-distribution ω ratio variation models**. The plots are derived from the estimated β-distribution parameters for the M7 (or M8, if statistically superior to M7) models for the NR genes and show for a particular gene how many codons have estimated ω ratios equal to or less than a particular ω ratio on the abscissa. In contrast to Figure 1, only data derived from analyses of all available species are included in Figure 2 (i.e., mammals-only comparisons are not included). **(A)**, **(B)**, and **(C) **apply to analyses of full-length sequences, DBD only, and LBD only, respectively. For part **(C)**, the red curve represents data for the LBD of the AHR gene. Analysis in part **(A) **is for NR1A1, 1B1, 1C1, 1F2, 1H3, 1I1, 1I2, 1I3, 2A1, 2B1, 3A1, 3A2, 3B1, 3C1, 3C3, 4A1, 5A1, 6A1, 0B1, and OB2; for part **(B)**, analysis is for NR1A1, 1B1, 1C1, 1H3, 1I1, 1I2, 1I3, 2A1, 2B1, 3A1, 3A2, 3B1, 3C1, 3C3, 4A1, 5A1, and 6A1; and for part **(C)**, analysis is for NR1A1, 1B1, 1F2, 1H3, 1I1, 1I2, 1I3, 2A1, 2B1, 3A1, 3A2, 3B1, 3C1, 3C3, 4A1, 5A1, 6A1, 0B1, OB2, and AHR.

Figure 2 shows plots derived from the estimated β-distribution parameters for the M7 (or M8, if statistically superior to M7) analyses for the NR genes. The β-distribution is a continuous function that for the PAML analysis is restricted to values between 0 and 1. Depending on the parameters α and β for the β-distributions, the values may be clustered more towards 0 or be more evenly distributed between 0 and 1. Figure 2 plots for a particular gene how many codons have estimated ω ratios equal to or less than a particular ω ratio on the abscissa. For example, for analysis of the LBD of NR3B1 (ERRα), the M7 analysis produces a β-distribution where all variation in ω ratios across codons is accounted for by ω ratios < 0.01 (see Figure 2C). In contrast, PAML analyses for the DBD and LBD of CAR show β-distributions that span a range of ω ratios greater than all other NR genes; the same analyses for PXR are second only to CAR in the range of ω ratios spanned (Figure 2B,C). The two LBD domain 'singletons', DAX-1 and SHP also show a wider span of ω ratios than most other NR genes (Figure 2A,C; DBD analysis for DAX-1 and SHP is not possible as these are domain singletons). Similar to the M0/M3 analyses described above, PAML M7/M8 analysis was also applied to the LBD of the AHR LBD. In contrast to PXR and CAR, the β-distribution of ω ratios across codons in the AHR LBD was more similar to the majority of NR genes than to PXR or CAR (see red line in Figure 2C).

### Individual variation among codons in selected NR genes

**Figure 3:**
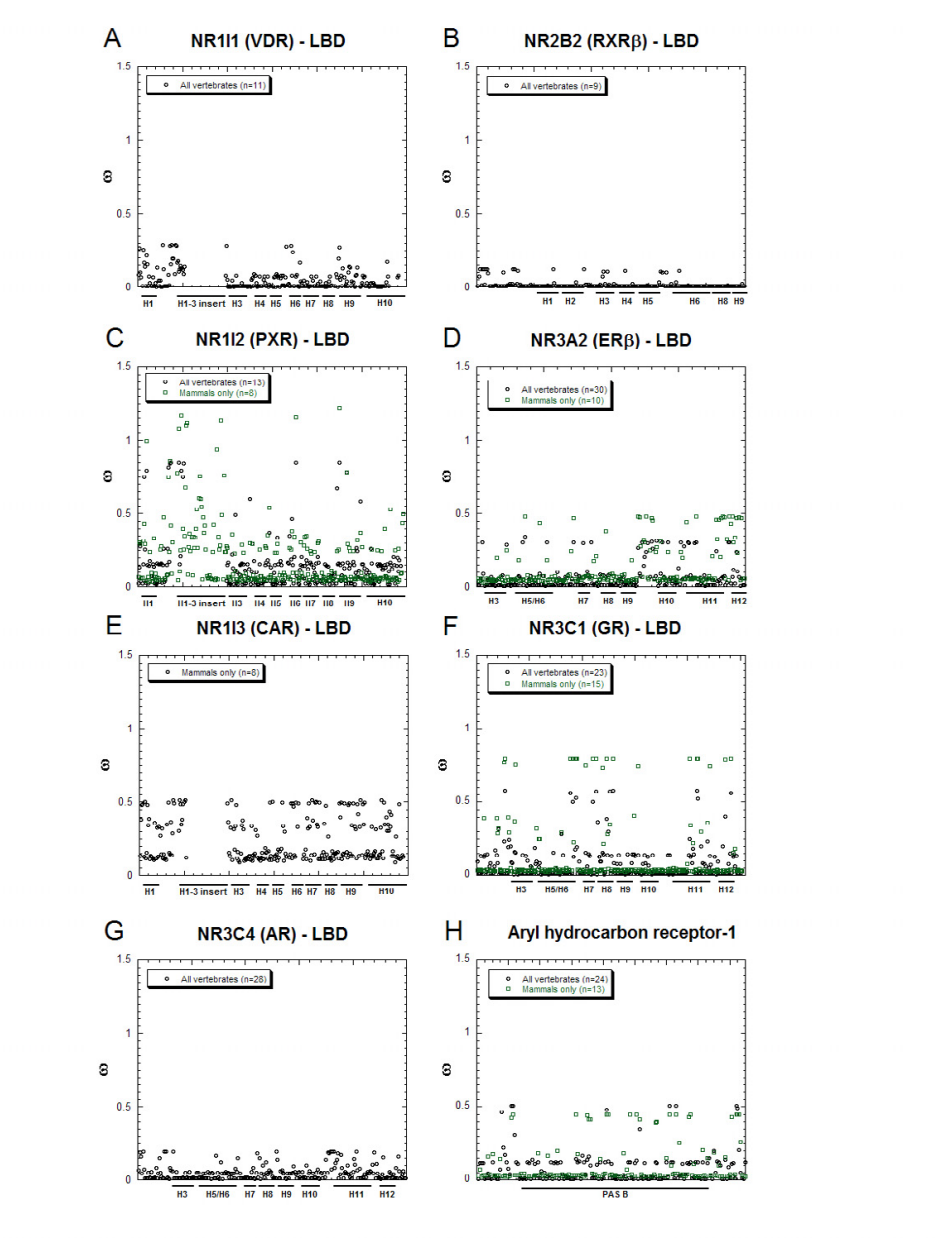
**Estimates of ω ratios for individual codons in the LBDs of 7 NR genes and the AHR gene**. The graphs in **(A) **through **(H) **plot the estimated ω ratios for individual codons of the LBDs of 7 NR genes and the AHR gene derived from the 'best minimum' PAML discrete model. The location of the α-helices in the LBDs of the NR genes that correspond to codons are indicated in the abscissas (e.g., 'H1' denotes α-helix-1; 'H1-H3 insert' denotes the insertion region in the NR1I subfamily proteins between helix-1 and helix-3); the location of the PAS-B domain is also shown for the AHR gene. CAR lacks the H1-H3 insert but this region is plotted in **(E) **to keep the alignment consistent between **(A) **VDR, **(C) **PXR, and **(E) **CAR. Due to difficulties in alignment and extreme sequence divergence for VDR and PXR in the H1-H3 insertion region, PAML analysis for this region could be performed for mammals only for the PXR genes. For NR1I1, NR2B2, and NR3C4, analysis restricted to mammals resulted in a best minimum PAML discrete model of only one ω ratio population (i.e., the M0 model); therefore, only data for all vertebrate species is plotted for those three genes (note also that the CAR gene is only found in mammals). The plots in **(A)**, **(C)**, and **(E) **show data for all three NR1I subfamily members and reveal that PXR has the widest variation of ω ratios across codons both within this subfamily (with CAR intermediate between PXR and VDR) and compared to the other NR genes.

The M3 PAML analyses also provide estimates of the mean ω ratio for each individual codon in a gene or gene domain. This provides some indication of which specific codons are potentially subject to positive selection. Figure 3 shows a plot of calculated mean ω ratios for the LBDs of 7 NR genes and the AHR gene. Comparison across the LBDs of NR1I subfamily genes shows that PXR has very diverse variation of ω ratios across codons with 5.1% of codons in the mammals only analysis having estimated mean ω ratios exceeding 1 (Figure 3C). In contrast, VDR, whose major function is to respond to 1,25-(OH)_2_-vitamin D_3 _(calcitriol), a ligand conserved across all vertebrate species, shows much lower inter-codon variation of ω ratios than CAR or PXR (compare Figures 3A,C,E). The pattern of ω variation across codons for CAR and the AHR gene are descriptively similar (Figure 3E,H). For the mammals-only analysis, higher mean ω ratios are clearly associated with the Helix1-3 (H1-3) 'insert' region of PXR. The H1-3 insert is very divergent across PXR genes and, similar to the CAR genes, the two *Xenopus laevis *BXRs lack this sequence entirely. This stretch of sequence was excluded from the analyses of VDR and PXR for all vertebrate species due to extreme sequence divergence and difficulties in alignment.

The analyses of other NR genes also show some variation of nucleotide diversity across different receptors. RXRβ (NR2B2) is illustrative of a group of NRs whose LBDs show very low ω ratios across all codons (Figure 3B). There are also differences between the LBDs of the sex and adrenocortical steroid receptors with the androgen receptor (AR, NR3C4) showing low ω ratios, the estrogen receptor-β (ERβ, NR3A2) somewhat higher, and the glucocorticoid receptor (GR, NR3C1) the highest ω ratios of the three (Figure 3D,F,G). The somewhat higher ω ratios for select codons in the GR may relate to this receptor likely being one of the evolutionarily 'newer' classical steroid receptors, the estrogen and progesterone receptors being the most ancestral [17-20].

The receptors described above are all ligand-dependent. A contrasting group of receptors are the 'ligand-independent' NRs, of which the estrogen-related receptors (ERRα, NR3B1; ERRβ, NR3B2; ERRγ, NR3B3) [35], steroidogenic factor 1 (SF-1; NR5A1) [36], and liver receptor homolog 1 (LRH-1, NR5A2) [36] are examples. These NRs are activated in the absence of ligand, although recent work has shown that phosphatidyl inositols are likely endogenous ligands for SF-1 and LRH-1 [36]. The evolutionary history of ligand-independent NRs is incompletely understood [11,20], with a proposal that ligand-binding is actually the ancestral state for SF-1 and LRH-1 [36]. The ω ratios for ERRα, ERRβ, ERRγ, SF-1, and LRH-1 were all on the lower end for the NR superfamily, both across all vertebrates and within mammals (Additional file 3: Results of PAML analysis and treefiles), with patterns of ω ratio variation across codons very similar to that seen with the androgen receptor (Figure 3G). While it is possible limited positive selection has occurred at a small number of codons in the five ligand-independent receptors discussed above, this was not detected by the PAML analysis.

**Figure 4:**
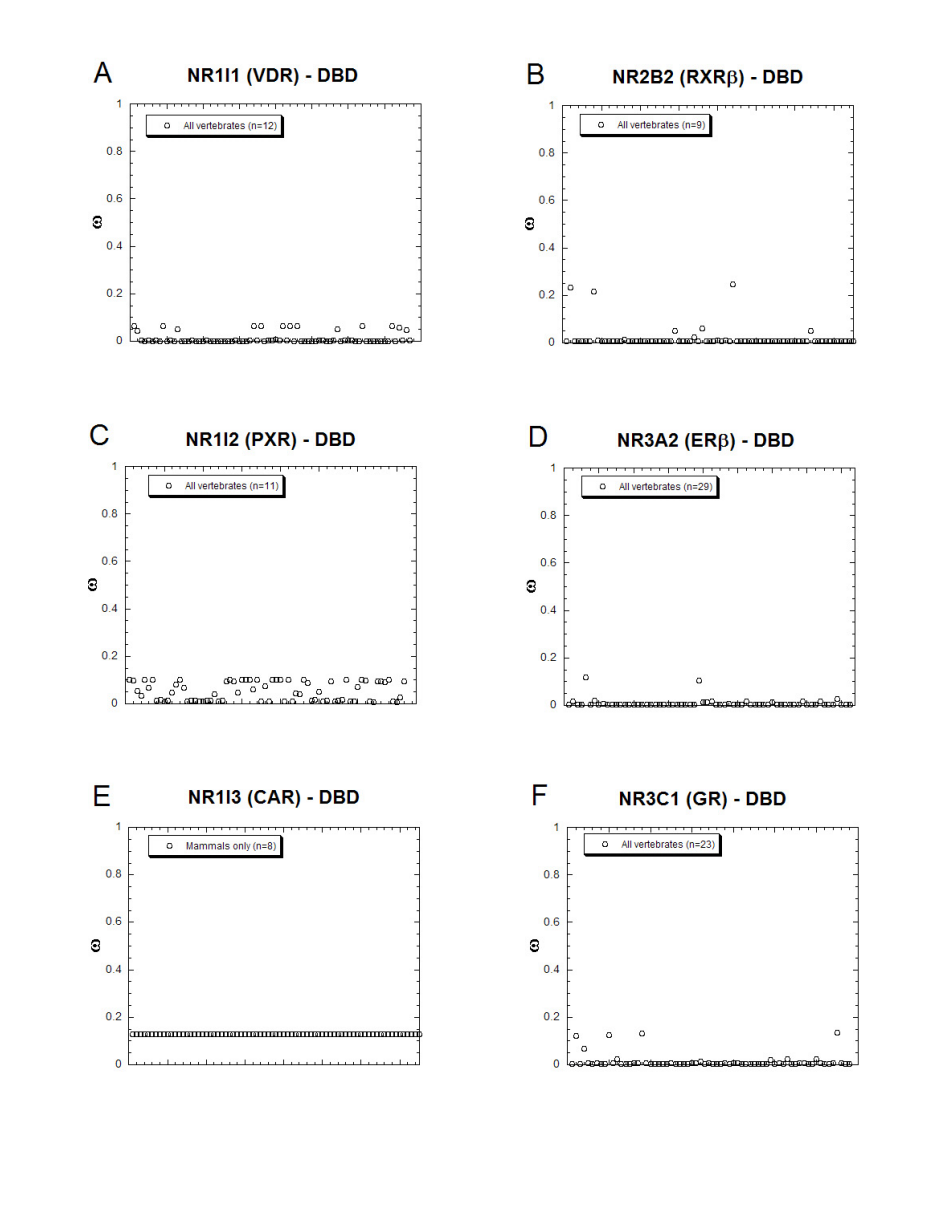
**Estimates of ω ratios for individual codons in the DBDs of 6 NR genes**. The graphs in **(A) **through **(F) **plot the estimated ω ratios for individual codons of the DBDs of 6 NR genes derived from the 'best minimum' PAML discrete model, utilizing sequence data from all available vertebrate species (note that the CAR gene is found only in mammals). In contrast to the analyses of the LBDs in Figure 3, the ω ratio variation in the DBDs for the six NR genes shown in **(A) **through **(F) **is limited and restricted to low ω ratios.

Figure 4 shows ω ratio variation across the codons of the DBDs of six NR genes. In general, the ω ratios are generally much lower than 1, consistent with strong purifying of the DBD. Overall, CAR and PXR show higher ω ratios across codons than the other four NR genes, but the calculated ω ratios are still less than 0.2 for all codons (Figure 4C,E).

### Comparisons of the NR1I subfamily genes

**Figure 5:**
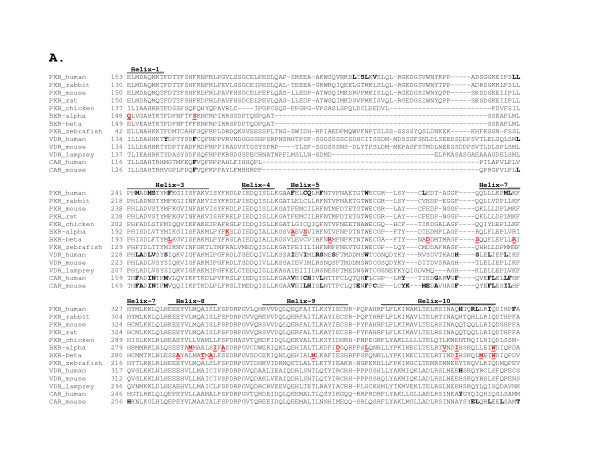
**Sequence alignment of the LBD of PXR, VDR, and CAR genes**. The locations of the α-helices above the amino acid sequences are based on the structures determined from x-ray crystallography of human PXR and human VDR [73, 88]. Amino acid resides highlighted in **bold **type are residues in human PXR, human VDR, mouse CAR, and human CAR shown to directly interact with structurally diverse ligands. These residues have been determined by x-ray crystallography and, in some cases, by additional molecular modelling for human VDR [39, 88-90], rat VDR [91], human PXR [37, 38, 73], mouse CAR [40, 92], and human CAR [81]. The ligands for the various receptors are: human VDR – calcitriol [39, 88, 89], 20-epi calcitriol analogs [89], calcipotriol, seocalcitol [39], 1α,25-lumisterol [90]; rat VDR – 2-carbon substituted vitamin D_3 _analogs [91]; human PXR – SR12813 [73], hyperforin [38], rifampicin [37]; mouse CAR – 5α-androst-16-en-3α-ol (androstenol) [92], TCPOBOP [40]; and human CAR – 5β-pregnan-3,20-dione and 6-(4-chlorophenyl)imidazo[2,1-b][1,3]thiazole-5-carbaldehyde *O*-(3,4-dichlorobenzyl)oxime (CITCO) [81]. The amino acid residues highlighted in **red underlined bold**in *Xenopus laevis *BXRα and BXRβ correspond to codons that show evidence of positive selection in a previously published phylogenetic analysis of nucleotide variation in the BXRα and/or BXRβ lineages [30]. Note that of the 23 amino acid residue positions identified as having high probability of having experienced positive selection in the BXRα and/or BXRβ lineages, 9 are orthologous to or adjacent to residues that are orthologous to human PXR residues shown to directly interact with the ligands SR12813, hyperforin, and/or rifampicin in x-ray crystallographic structures of the human PXR [37, 38, 73]; an additional two residues are orthologous to ligand-binding residues in human VDR [39, 88-90] and, also in one case, human and mouse CAR as well [40, 81, 92].

The NR1I subfamily includes VDR, PXR, and CAR. Sequence alignments of the LBDs of selected genes from this subfamily are presented in Figure 5. Amino acid residues identified as directly interacting with ligands in high-resolution crystal structures are indicated in **bold **type. As can be seen from inspection of Figure 5 and highlighting the shared history of PXRs, CARs, and VDRs, a number of orthologous amino acid residues are involved in ligand binding at more than one receptor. For example, leucine-240 of the human PXR directly interacts with the antibiotic rifampicin [37] and hyperforin (active component of the herbal anti-depressant St. John's wort) [38]; the orthologous residue in human VDR (L227) directly interacts with calcitriol analogs [39] while the same position in mouse CAR (L168) binds the pesticide contaminant 1,4-bis [2-(3,5-dichloropyridoxyl)]benzene (TCPOBOP) [40].

In a previous publication, the authors have determined codon positions in the *Xenopus laevis *BXRs that show evidence for positive selection in the evolution of this unusual lineage of PXRs; 23 such codons were identified, and all were located in the LBD of the BXRs [30]. The BXRs are notable in the PXRs for having *lost *the ability to be activated (and presumably to bind) structurally diverse ligands. In addition, these receptors have a tissue expression pattern markedly different than other PXRs, being found at high levels in gonadal tissue but not xenobiotic-metabolizing organs such as liver or the intestines [41-43], and are activated efficaciously only by benzoate compounds that have a role in frog development [41,42,44]. Interestingly, 9 of the 23 amino acid residue positions in the BXR lineages that have evidence of positive selection are orthologous to or directly adjacent to residues that are orthologous to human PXR residues shown to directly interact with the ligands SR12813, hyperforin, and/or rifampicin in x-ray crystallographic structures of the human PXR (Figure 5); an additional two residues are orthologous to ligand-binding residues in human VDR and, also in one case, to ligand-binding residues in human and mouse CAR as well. The evidence is consistent with positive selection in the evolution of the BXRs being directed at the LBD to alter ligand specificity, in large part by targeting amino acid residues capable of directly interacting with ligands. This has presumably been a significant factor underlying the much narrower ligand selectivity observed in the modern BXRs.

**Figure 6:**
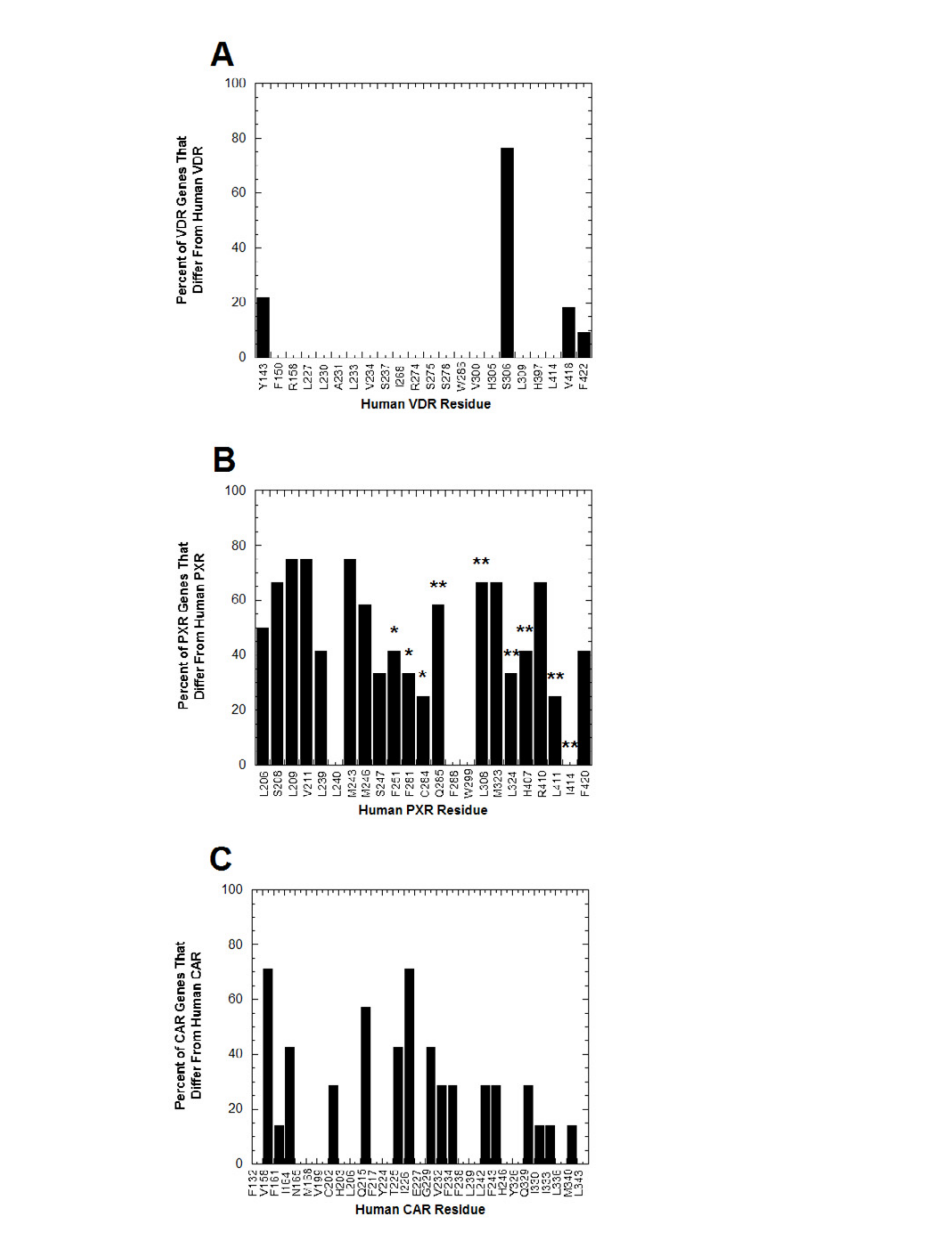
**Conservation of ligand-binding residues in VDR, PXR, and CAR**. From high-resolution, x-ray crystallographic structures of human VDR, rat VDR, human PXR, mouse CAR, and human CAR bound to various ligands, the amino acid residues that directly interact with ligands are known (see Figure 5; also see Additional file 1: Genes used for phylogenetic analysis for complete list of species available and their accession numbers). **(A) **Of the 22 amino acid residues shown to interact with ligands at human and/or rat VDRs, only 4 residues show any sequence variation across vertebrate species. The remaining 18 of 22 residues show complete conservation across all vertebrate VDRs (from sea lamprey to human VDRs). Eighteen VDRs were used for the analysis. Due to partial sequence, data for the chimpanzee VDR was only available for the first two ligand-binding residues (corresponding to human VDR Y143 and F150); in addition, data was missing for the four most C-terminal ligand-binding residues (corresponding to human VDR H397, L414, V418, and F422) for crocodile, snake, turtle, lizard, frog, and fugu-β VDRs. **(B) **In contrast to the VDRs, the PXRs show extensive amino acid sequence divergence at the residues shown to interact with ligands at the human PXR. Only 3 of 23 positions are conserved throughout the 13 vertebrate PXRs while for 9 of 23 residues, over half of the PXRs have an amino acid residue that differs from that at the human PXR. Also indicated are the 9 amino acid residues in the BXRα and/or BXRβ lineages that show evidence for positive selection (see Figure 5 legend for more details; * indicates BXRα and/or BXRβ residue directly orthologous to human PXR ligand-binding residue; ** indicates residue adjacent to such a ligand-binding residue). **(C) **CARs also show much more divergence at ligand-binding positions than human VDR but not as great as the PXRs. The data is based on eight complete mammalian CAR sequences.

The NR1I subfamily members also differ markedly in conservation of ligand-binding residues across vertebrate species. Figure 6 shows for VDRs, PXRs, and CARs how many species differ from the human receptor at the amino acid residue positions known to interact directly with ligands. VDRs show tight conservation of ligand-binding residues. Only 4 of 22 amino acid residues show any variation at all across species, ranging from jawless fish (sea lamprey), teleost fish, reptiles, frog, birds, and mammals (Figure 6A). PXRs, on the other hand, show extensive amino acid sequence divergence at ligand-binding residues with only 3 of 23 positions conserved across all 13 vertebrate PXRs currently sequenced; for 9 of 23 positions, over half of the non-human PXRs have an amino acid residue different from the human sequence (Figure 6B). CARs also show more divergence at ligand-binding positions than human VDR but not as great as the PXRs, although the analysis is limited due to the presence of CAR genes only in mammals (Figure 6C).

#### Functional studies of VDRs and PXRs and a proposed phylogeny of the NR1I subfamily

Previous studies have revealed the broad ligand specificity of mammalian and chicken PXRs; these receptors are activated by a structurally diverse array of xenobiotics and endogenous compounds including bile salts, steroid hormones, and prescription drugs [30,44-50]. A recent study has revealed that while zebrafish PXR does not respond to mammalian 24-carbon (C_24_) bile acids such as cholic acid, chenodeoxycholic acid, or lithocholic acid, this PXR is activated well by the zebrafish 27-carbon (C_27_) bile alcohol sulfate (cyprinol sulfate) [30], a biliary bile salt typical of the earliest biliary detergents to evolve in vertebrates [51]. VDRs, on the other hand, have a much more restricted ligand specificity, having adapted to bind calcitriol at nanomolar or subnanomolar affinity [52]. Mammalian VDRs do, however, have the ability to be activated by the toxic secondary bile salt lithocholic acid and its metabolites, a function that confers a protective role in the intestine against this toxic secondary bile acid [53].

**Figure 7:**
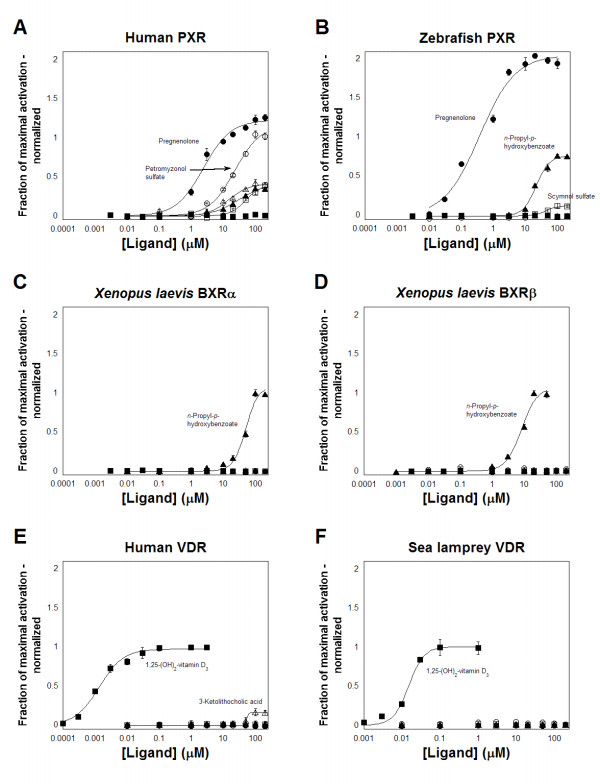
**Concentration-response curves of activation of PXRs and VDRs by endogenous ligands or their analogs**. The ordinate represents activation of the PXR or VDR, relative to vehicle control, and normalized to the maximal activator (rifampicin for human PXR, 5α-androst-3α-ol for zebrafish PXR, *n*-butyl-*p*-aminobenzoate for *Xenopus laevis *BXRα, *n*-propyl-*p*-hydroxybenzoate for *Xenopus laevis *BXRβ, and calcitriol for human and sea lamprey VDRs; see Materials and Methods for more details). The drugs tested were pregnenolone (●), petromyzonol sulfate (sea lamprey bile salt; ○), scymnol sulfate (cartilaginous fish bile salt; □), 3-ketolithocholic acid (mammalian bile acid metabolite; △), *n*-propyl-*p*-hydroxybenzoate (▲), and 1,25-(OH)_2_-vitamin D_3 _(calcitriol; ■). **(A) **Human PXR is activated by micromolar concentrations of the steroid pregenonolone, the early bile salts petromyzonol sulfate and scymnol sulfate, 3-ketolithocholic acid, and the benzoate analog. Calcitriol does not activate human PXR. **(B) **Similar to human PXR, zebrafish PXR is activated by the steroid pregnenolone, the cartilaginous fish bile salt scymnol sulfate, and the benzoate analog, but not by the other compounds. **(C, D) **The *Xenopus laevis *BXRs do not share any ligands with human and zebrafish PXRS other than the benzoate analog *n*-propyl-*p*-hydroxybenzoate, which activates BXRα and BXRβ robustly. **(E, F) **Human and sea lamprey VDRs are both activated robustly by nanomolar concentrations of calcitriol. Similar to human PXR, 3-ketolithocholic acid activates human VDR at micromolar concentrations, with an efficacy of only 15% relative to calcitriol. Sea lamprey was not activated at all by 3-ketolithocholic acid. Weak concentration-dependent activation of sea lamprey VDR by petromyzonol sulfate (○) was observed; however, the efficacy of this bile salt was only ~5–6% relative to that of calcitriol. In panels **(A, E, F)**, full-length receptors for human PXR, human VDR, and sea lamprey VDR were used, with the reporter plasmid being CYP3A4-PXRE-Luc. In panels **(B, C, D)**, GAL4-LBD fusion constructs were used for zebrafish PXR and the *Xenopus laevis *BXRs, with the reporter plasmid being tk-UAS-Luc.

Figure 7 examines the response of four PXRs and two VDRs to pregnenolone (a pregnane steroid), scymnol sulfate (a C_27 _bile alcohol sulfate from cartilaginous fish), petromyzonol sulfate (an unusual C_24 _bile alcohol sulfate from the sea lamprey), 3-ketolithocholic acid (a metabolite of lithocholic acid), calcitriol, and a benzoate analog (*n*-propyl-*p*-hydroxybenzoate). Human PXR is activated by micromolar concentration of all 6 compounds except calcitriol (Figure 7A). As previously proposed [30], activation of human PXR by 'early' bile salts such as petromyzonol sulfate and scymnol sulfate likely represents an 'ancestral' property retained in mammalian PXRs (in contrast, unconjugated scymnol, the precursor to the excreted scymnol sulfate, was inactive at all receptors tested). Similar to human PXR, zebrafish PXR is activated by pregnenolone, scymnol sulfate, and the benzoate analog, but not by the other compounds (Figure 7B). The lack of activation of zebrafish PXR by petromyzonol sulfate may be a result of this compound being an unusual C_24 _bile alcohol sulfate. The sea lamprey has apparently independently evolved the ability to cleave the cholesterol side-chain because C_24 _bile alcohol sulfates are not found in other fish, and the peroxisomal mechanisms used to cleave the cholesterol side-chain of C_24 _bile acids evolved more recently than our last common ancestor with lampreys [51,54]. The *Xenopus laevis *BXRs do not share steroid or bile salt ligands with human or zebrafish PXR, but are activated well by the benzoate analog (Figure 7C, D).

Despite over 500 million years since the last common ancestor of humans and jawless fish, the human and sea lamprey VDRs are activated very similarly by calcitriol (Figure 7E, F). The major difference in ligands between these two receptors is the activation of human VDR by lithocholic acid and its metabolites (Figure 7E)[30,53]. Of the six compounds tested, only calcitriol activated the sea lamprey VDR efficaciously. The sea lamprey biliary surfactant petromyzonol sulfate produced a weak but concentration-dependent activation that had a maximal effect only 5–6% that of calcitriol (Figure 7F). The possible physiologic significance of this weak *in vitro *effect needs to be correlated with *in vivo *experiments in sea lampreys.

Overall, VDRs are activated by nanomolar concentrations of calcitriol, a ligand that does not activate PXRs. In contrast, zebrafish PXR and human PXR share similar activation by pregnane and androstane steroids along with early bile salts, as indicated by other studies [30,44]. Recent work has also revealed that activation of the zebrafish PXR *in vivo *upregulates the expression of cytochrome P450 (CYP) 3A and multi drug resistance 1 (MDR1) genes [55], properties shared by other PXRs (except in the frog) [46,49,56,57].

## Discussion

The search for genes that show evidence for positive Darwinian selection has been an important focus of molecular phylogenetics [21,22]. The genes in the NR superfamily generally show nucleotide variation across species consistent with strong purifying selection, particularly in the DBDs. This study applied phylogenetic analysis by a maximum likelihood method to the entire NR superfamily in vertebrates to analyze patterns of nucleotide variation that may be suggestive of positive selection. The results extend previous more limited phylogenetic analysis of PXR and CAR genes [8,29,30,58] and clearly show that the LBDs of the PXR and CAR genes have ω ratios at the extreme high end for the NR superfamily. Two other genes that have similar variation in ω ratios are DAX-1 and SHP, two NRs that lack a defined DBD and are classified as LBD singletons [12,13]. The elevated ω ratios in DAX-1 and SHP, relative to other NR genes, may be related to the unique evolutionary pressures these two genes face as domain singletons [8,11].

This possible signature of positive selection in the LBDs of the PXR and CAR genes is consistent with the role of PXR and CAR as sensors of toxic endogenous and exogenous compounds that may vary across species [5,6]. For PXR and CAR, evolutionary selection would be directed at fine-tuning ligand specificity towards the most important toxic compounds (or class of compounds) for a given species. PXR and CAR clearly differ markedly in nucleotide variation from VDR, the other member of the NR1I subfamily; in terms of nucleotide variation, VDR behaves like other NRs and not PXR or CAR. The elevated ω ratios of PXR and CAR are not simply a result of synonymous and non-synonymous substitutions being increased in tandem. As with two gene comparisons within mammals [8], synonymous substitutions rates for PXR and CAR genes across vertebrates are average when compared to other NR genes [30,58]. The estimated ω ratios for the codons of the LBDs of CAR and PXR genes are also higher than those for the AHR gene, a non-NR that qualitatively has very similar functions to CAR and PXR, including the ability to upregulate the expression of CYP genes involved in detoxification of xenobiotics [34].

An additional finding with CAR was that the ω ratios for the CAR DBD are on the higher end for the NR superfamily. This may be a reflection of the recent divergence of the CAR gene following duplication of a pre-mammalian PXR gene [59]. Functional diversification with higher rates of non-synonymous to synonymous substitutions is common in a gene that has recently duplicated [60,61]. Once two copies of a gene exist, one of the two genes is free to diversify function to evolutionary advantage although the two genes still may share overlapping functions. The CAR and PXR DBDs show considerable cross-reactivity with one another in terms of interactions with target gene response elements [62-67], extending even to the chicken PXR [68]. This cross-reactivity highlights the close evolutionary history of PXR and CAR and may indicate that CAR provides physiologically important redundancy to important functions of PXR in mammals. (Note that even PXR and VDR, which are more distantly related than CAR and PXR, show considerable overlap in target gene regulation. VDR is able to upregulate expression of detoxifying proteins such as CYP3A4 [53,69,70] while PXR has recently been shown to regulate expression of proteins involved in the metabolism of calcitriol and related molecules [71,72]). The relatively high ratio of non-synonymous to synonymous substitutions of the CAR DBD (at least as compared to other NRs) suggests, however, that the CAR DBD is not evolutionarily 'static' and is diversifying to recognize other binding elements or to interact differently than PXR with shared binding elements.

The phylogenetic analyses presented here do have some limitations. The number of sequences available for analysis varies markedly across the NR superfamily. For those genes that have few sequences available, the phylogenetic analysis will have more limited power to detect small sub-populations of codons with elevated ω ratios [22,32,33]. On the other hand, analysis of too few genes runs the risk of increasing false positives [22,31-33]; this was the reason a minimum of number of six genes from six different genera was applied to the NR superfamily dataset. In addition, difficulties in sequence alignment are a potential problem for some genes and necessitated the removal of some codons from phylogenetic analysis. For the NR1I subfamily, this was particularly an issue with the H1-H3 insert. This sequence was not analyzed for the PXR and VDR genes when applied to all vertebrates. Interestingly, the H1-H3 insert region of PXR genes within mammals was identified as a region where a number of the codons have estimated ω ratios greater than one. Given the role of this region in expanding the ligand-binding pocket of human PXR relative to other NRs [37,38,73], variation in the H1-H3 insert region within mammalian PXRs may have allowed for evolutionarily advantageous changes in ligand specificity across species.

**Figure 8:**
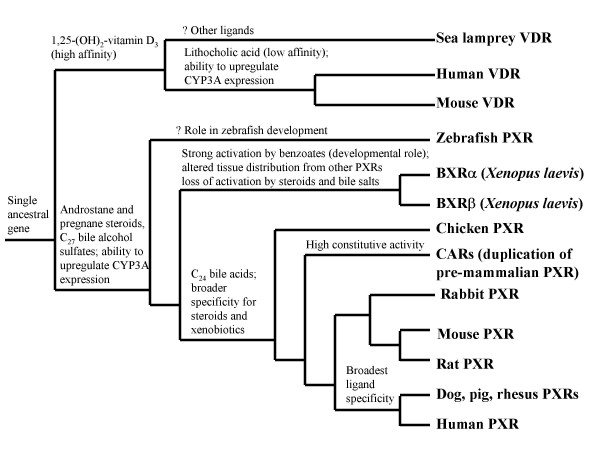
**Proposed phylogeny of the NR1I subfamily**. The phylogenetic tree is based on known phylogenetic relationships between the species combined with functional data from this study and others. Features included are activation by pregnane and androstane steroids, C_27 _bile alcohol sulfates (like cyprinol and scymnol sulfate), C_24 _bile acids (such as cholic and lithocholic acid), benzoates, and calcitriol; ability to increase (upregulate) the expression of the CYP3A enzymes; and high constitutive (baseline) activity. It is possible that activation of PXRs by benzoates is an ancestral property as all PXRs can be activated by at least some benzoate compounds [44], although functional roles of these compounds have so far only been demonstrated in frogs [41, 42]. The study of ligand effects on CARs is complicated by the high constitutive activity of these receptors; many ligands act as inverse agonists of CARs. The possible developmental role of zebrafish PXR is highlighted by its strong expression in early life stages of zebrafish [74].

Figure 8 shows a proposed phylogeny of the NR1I subfamily, taking into account data from this study and others. The evolutionary history of PXR and CAR has not been fully resolved. Experimental analysis and ongoing genome sequencing projects have not revealed a CAR gene in teleost fish, with zebrafish, *Fugu rubripes*, and *Tetraodon nigroviridis *each possessing a single gene classified as a PXR due to closer sequence and functional similarity to mammalian PXR genes than CAR genes [9,44]. Similarly, the chicken possesses only a single PXR/CAR-like gene (the 'chicken X receptor' or CXR) with sequencing of the chicken genome, extensive attempts to clone an additional NR1I receptor gene, and RNA interference of the CXR gene coupled with functional assays failing to show direct or indirect evidence of an additional NR1I subfamily gene member [59]. Unlike telost fish PXRs, the CXR has equal sequence similarity to mammalian PXRs and CARs and shares a number of properties with mammalian CARs, including high constitutive activity in *in vitro *assays and lack of sequence in the H1-H3 insert region, leading to the possibility that the CXR gene is actually a CAR gene and not a PXR gene as currently classified [44,48,59]. The phylogeny in Figure 8 classifies the CXR as a PXR and presents the parsimonious explanation that fish, birds, and their ancestors possessed a single PXR gene that duplicated in an ancestral mammal or pre-mammal. One of the two resulting genes then diverged to the CAR gene [59]. This hypothetical phylogeny will be strengthened or modified by analysis of PXR/CAR genes in reptiles and additional mammals such as marsupials or monotremes.

As described above, the *Xenopus laevis *BXRs are an especially intriguing example of extensive divergence of a gene from other vertebrate orthologs. The BXRs have dramatically altered ligand specificity and tissue expression relative to other PXRs. The BXR lineage shows strong evidence of positive selection [30], directed particularly at likely ligand-binding residues, based on comparison to known ligand-binding residues of human PXR, human VDR, mouse CAR, and human CAR.

An unanswered question in the NR1I subfamily is the functional properties of the closest common ancestor to PXR and VDR. Clues to this may be revealed if PXR can be cloned and characterized from a jawless fish such as lampreys or hagfish or with exploration of invertebrate ortholog(s) to these receptors. Sequencing of the genome of the chordate *Amphioxus*, one of the closest invertebrate relatives to vertebrates, and sea lamprey may be insightful in this regard. There are suggestions that zebrafish PXR may have important roles in early zebrafish development [74], which, if true, would provide a functional link between the zebrafish PXR and the frog BXRs [41,43].

The presence of a high-affinity VDR in the sea lamprey, a jawless fish with a non-calcified cartilaginous skeleton, suggests that regulation of calcium and phosphate levels was not a function (or a critical function) of the 'ancestral' VDR [75]. The high concentrations of calcium in sea water mean that maintaining adequate calcium levels in tissues and body fluids is mainly a problem for terrestrial or fresh water animals. In mammals, VDRs mediate a number of functions other than regulation of calcium and phosphate levels, with critical roles in the immune system and skin development [76,77]. *Ciona intestinalis *(sea squirt), a urochordate that is the closest invertebrate relative to vertebrates for which relatively complete genome information is available, has a single NR equally related to VDR/PXR/CAR and a *Drosophila melanogaster *NR [78]. The properties of this invertebrate gene remain uncharacterized. These findings highlight how little is known of the physiology of early fish and chordate invertebrates and of the role of NRs in these animals.

The marked diversity of the PXR and CAR LBDs across vertebrates contrasts markedly with detailed resequencing studies of the human PXR and CAR genes that reveal that non-synonymous mutations in the LBDs of these genes are rare. Resequencing of 70 individuals from three different ethnic groups for the CAR gene [58] and approximately 100 individuals from several different ethnic groups for the PXR gene [79] showed very low nucleotide diversity and no nonsynonymous substitutions in the LBD of either gene. Sequencing of 253 Japanese subjects revealed only a single non-synonymous substitution in the CAR LBD [80]; the residue identified (valine-133) is adjacent to a ligand-binding residue of human CAR [81] and mouse CAR [40]. Sequencing of 205 Japanese subjects found two non-synonymous PXR substitutions (R381W and I403V) as single alleles in separate individuals [82]; these mutations caused modest reductions in transactivation of a CYP3A4-based reporter [83]. Another PXR re-sequencing study found a single D163G substitution in 1/74 Africans and 0/418 Caucasians and a single A370T substitution in 1/64 Africans and 0/312 Caucasians [84]. In addition, the nucleotide divergence of the human and chimpanzee CAR and PXR genes are lower than the average for other genes in the human genome [29,58,85]. This suggests that important functions of the LBDs of PXR and CAR, including ligand specificity, do not vary significantly across human populations, and perhaps not between chimpanzees and humans as well, but do vary between humans and other mammals. Future studies should identify the ligands that have shaped the variation of PXR and CAR across species.

## Conclusion

NR genes generally show strong sequence conservation and little evidence for positive selection. The main exceptions are PXR and CAR, genes that may have adapted to cross-species differences in toxic compound exposure. Future studies will be directed at precisely defining the cross-species structural variation in CARs and PXRs and relating this to evolutionarily relevant differences in toxic compound exposure.

## Methods

### Phylogenetic analysis

Sequences for NR genes were downloaded from public databases National Center for Biotechnology Information (NCBI; ) and Ensembl . Complete listing of all genes, species, and accession numbers are provided in Additional file 1: Genes used for phylogenetic analysis. Fragmentary sequences missing 10 or more codons were excluded from the analysis. In situations where supporting functional data was not available, orthology was confirmed by reciprocal BLAST searches. Sequences were aligned with Clustal X. Regions of sequences that could not be aligned between species were excluded from analyses. This was primarily an issue when attempting to align certain non-mammalian NRs with mammalian NRs, a difficult problem especially for the PXRs [30,44]. Estimation of d_N_/d_S _(ω) ratios was carried out by maximum likelihood using a codon-based substitution model in PAML (Phylogenetic Analysis by Maximum Likelihood) version 3.13 [25,26,28]. The input to PAML is a treefile of the phylogeny of the sequences to be studied and a file with aligned sequences (see Additional file 2: Sequences used for phylogenetic analysis by PAML and Additional file 3: Results of PAML analysis and treefiles). The phylogeny is based on known phylogenetic relationships between the species to be studied, determined by a consensus of morphological and molecular data [86]. The treefiles for all analyses are in Additional file 3: Results of PAML analysis and treefiles.

PAML determines estimates of ω ratios for models of varying complexity. The most commonly applied models are as follows (the PAML model numbers are shown in parestheses; the 'sites' refers to codons) [25,26]: model M0 (null model with a single ω ratio among all sites), M3 ("discrete" model, with 2 or more categories of sites with the ω ratio free to vary for each site at any value from 0 to greater than 1). M7 ("β model", ten categories of sites, with ten ω ratios in the range 0–1 taken from a discrete approximate of the β distribution), and M8 ("β plus ω " model, ten categories of sites from a β distribution as in M7 plus an additional category of sites with an ω ratio that is free to vary from 0 to greater than 1). PAML estimates the ω ratios that are allowed to vary under these models, as well as the proportion of sites (codons) with each ratio.

Of the PAML models listed above, M0, M3 and M8 can detect positive selection (i.e., ω > 1), although it would be unlikely that M0 would show ω > 1 for any NR gene given the rarity of such high ω ratios across all codons of an entire vertebrate gene or gene domain [24]. Each PAML model generates a log-likelihood, indicating how well the model fits the input data. Some PAML models are "nested" within each other (e.g., M0 within M3, M7 within M8). In those cases, twice the log-likelihood difference between the two models is compared with a X^2 ^distribution with degrees of freedom equal to the difference in degrees of freedom between the two models [M0 has 1 degrees of freedom; M3 with two categories of ω sites (defined in the PAML software as ncatG = 2) has 3 degrees of freedom; M3, ncatG = 3, has 5 degrees of freedom; M3, ncatG = 4, has 7 degrees of freedom; M7 has 2 degrees of freedom; M8 has 4 degrees of freedom] [25,26]. *P *values for sites potentially under positive selection are obtained using a Bayesian approach in PAML [87]. The accuracy and power of PAML models increases with more sequences and longer length sequences [32,33]. Analyses were only performed if at least six species from six separate genera were available. Below six taxa, the power of PAML to detect positive selection is limited, and the risk of false positives increases [22,31-33]. Simpler PAML models are preferred unless a more complex model fits the data significantly better. Data from a more complex PAML model is presented only if twice the log-likelihood difference between that model and the closest simpler model (e.g., M0 compared with M3, ncatG = 2; M7 with M8) differs significantly with a *P *< 0.05 according to a X^2 ^distribution.

### Functional assays of PXR and VDR

Ligand activation of PXRs and VDRs was determined by a luciferase-based functional assay using methods previously described [30]. Briefly, HepG2 (human liver) cells stably expressing human Na^+^-taurocholate cotransporter (NTCP; SLC10A1) were used [30]. For experiments involving sulfated bile salts, human OATP (OATP; SLC21) was co-transfected at 10 ng/well to facilitate bile salt uptake. Mouse VDR (IMAGE clone 3710866) and pCMV-sport6 vectors were obtained from Invitrogen (Carlsbad, CA, USA). The zebrafish RXRβ cDNA clone (IMAGE clone 5410111) was obtained from ATCC (Manassus, VA, USA). The expression vectors were either full-length receptors (i.e., containing both a DBD and LBD; human PXR, human VDR, mouse VDR, sea lamprey VDR) or GAL4/PXR chimeras that contain only the LBD of the PXR receptor (BXRα, BXRβ, and zebrafish PXR). For the full-length expression vectors, the reporter plasmid was CYP3A4-PXRE-Luc, a construct that contains a promoter element from CYP3A4 (recognized by PXR and VDR DBDs) driving luciferase expression. For the GAL4/LBD expression constructs, the reporter plasmid was tk-UAS-Luc, which contains GAL4 DNA binding elements driving luciferase expression. The sea lamprey VDR cDNA was co-transfected with zebrafish RXRβ (15 ng/well) for more robust expression [30,75].

It should be pointed out that cross-species differences in the DBDs of various PXRs could impact the ability of a particular PXR to activate the human CYP3A4-based promoter driving luciferase expression. However, this is unlikely to affect the pharmacology of the various ligands studied in this report, particularly as ligand activation of a particular receptor was normalized to a specific maximal activator. The most distantly related PXRs to the human PXR, zebrafish PXR and frog BXRα and BXRβ, were studied using GAL4-LBD fusion constructs, so issues of cross-species differences in the DBD do not affect those receptors in this study. Although the sea lamprey is evolutionarily distant from mammals, the full-length sea lamprey VDR robustly activates the CYP3A4-PXRE-Luc reporter in response to calcitriol.

Activation of receptor by ligand was compared to receptor exposed to identical conditions without ligand ('vehicle control'). In general, dimethyl sulfoxide (Sigma) was used as vehicle and was adjusted to be 1% (v/v) in all wells. A control was also run with transfection of 'empty' vector (i.e., lacking the receptor cDNA) and reporter vector to control for activation of reporter vector by endogenous receptor(s). In experiments with a variety of activators, activation by endogenous receptors was not seen. Experiments were performed in quadruplicate and repeated for a total of at least three times. Concentration-response curves were fitted using Kaleidagraph software (Synergy Software, Reading, PA, USA). Data are presented throughout as mean ± S.E.M. In combining data from multiple experiments, the pooled variance was calculated by the formula s_pooled _= {[(n_1_-1)s_1_^2 ^+ (n_2_-1)s_2_^2 ^+ ... + (n_k_-1)s_k_^2^]/[N-k]}}^-1/2^, where there are N total data points among k groups, with n replicates in the i^th ^group.

Each PXR or VDR construct was tested with compounds previously shown to be robust activators of the respective receptors [30]. To facilitate more reliable cross-species comparisons, complete concentration-response curves for ligands were determined in the same microplate as determination of response to a maximal activator. This allows for determination of relative efficacy, ε, defined as the maximal response to test ligand divided by maximal response to a reference maximal activator (note that ε can exceed 1). Compounds with ε < 1 were considered 'inactive.' The maximal activators and their concentrations for the PXRs and VDRs studied are as follows: human PXR – 10 μM rifampicin; zebrafish PXR – 20 μM 5α-androstan-3α-ol; *Xenopus laevis *BXRα – 30 μM *n*-butyl-*p*-aminobenzoate; *Xenopus laevis *BXRβ – 50 μM *n*-propyl-*p*-hydroxybenzoate; human VDR – 1 μM calcitriol; and sea lamprey VDR – 0.3 μM calcitriol.

Scymnol sulfate was isolated from bile of the Spotted eagle ray (*Aetobatus narinari*) by extraction and Flash column chromatography. Scymnol sulfate was deconjugated using a solution of 2,2-dimethoxypropane:1.0 *N *HCl, 7:1 v/v, and incubating 2 hours at 37°C, followed by the addition of water and extraction into ether. Completeness of deconjugation and assessment of purity was performed by thin-layer chromatography using known standards. Other bile salts and steroids were from Steraloids (Newport, RI, USA). All other chemicals were from Sigma (St. Louis, MO, USA).

## Competing interests

The author(s) declare that they have no competing interests.

## Authors' contributions

M.D.K. conceived of the study, carried out the majority of experiments, and drafted the manuscript. K.Y. contributed to the experiment, performed some of the cloning and molecular biology, and generated the stable HepG2 cell line used for functional assays. E.G.S. participated in the design of the study and helped edit the manuscript. L.R.H. isolated and purified the cartilaginous fish bile salt and helped edit the manuscript. All authors read and approved the final manuscript.

## Supplementary Material

Additional File 1 **Genes used for phylogenetic analysis**. Excel file containing accession numbers for all files used in the phylogenetic analysis. Click here for file

Additional File 2 **Sequences used for phylogenetic analysis by PAML**. Text file with complete, full-length nucleotide sequences for all genes used in the PAML analysis. The nucleotide numbers corresponding to the DBD and LBD are also indicated. Click here for file

Additional File 3 **Results of PAML analysis and treefiles**. Excel file containing summary data for all PAML analyses. The log-likelihood for each analysis is given along with additional relevant summary parameters. The methods for statistically comparing nested models are described in Materials and Methods. In addition, the phylogenetic treefile for each analysis is also included. Click here for file
